# Surgeon St. George Peachy

**Published:** 1865-02

**Authors:** 


*Surgeon St. George Peachy.*

Died, at his residence in this city, December 30th, 1864, after a long illness, Surgeon St. George Peachy, in the 36th year of his age.

Though long expected, this event is none the less painful, for however calmly we may consign age and affection to its last resting place, early life, with all its aspirations, has a hold upon our hopes and affections which we cannot so easily sever. Gaps in the social and professional circle may be filled by the cumulus of time, but to those from whose hearts he is rent, and coming in these troublous times, even while affliction had just passed out and left the gates of sorrow ajar, the loss is terrible indeed. Theirs is the grief that sympathy may soften but not abridge. May the hand that tempers the wind deal gently with their wounds.

Dr. Peachy had attained to enviable eminence in his profession, and leaves behind a lesson of skill, ability, and zeal, by which those that come after him may well profit. Since the beginning of the war his energies had been almost exclusively devoted to the medical business of the army, and doubtless it was his vigorous and industrious intellect dragging an unequal body to its tasks that helped to seal his early career.

"Requiescat in pace."

